# Public Knowledge and Practices Regarding Coronavirus Disease 2019: A Cross-Sectional Survey From Pakistan

**DOI:** 10.3389/fpubh.2021.629015

**Published:** 2021-04-26

**Authors:** Muhammad Saqlain, Ali Ahmed, Ijaz Nabi, Aqsa Gulzar, Sahar Naz, Muhammad Muddasir Munir, Zaheer Ahmed, Sohail Kamran

**Affiliations:** ^1^Department of Pharmacy, Quaid-I-Azam University, Islamabad, Pakistan; ^2^School of Pharmacy, Monash University, Bander Sunway, Malaysia; ^3^Faisalabad Medical University, Faisalabad, Pakistan; ^4^School of Pharmacy, Monash University, Bander Sunway, Malaysia; ^5^Institute of Pharmacy, Lahore College for Women University, Lahore, Pakistan; ^6^School of Pharmacy, The University of Faisalabad, Faisalabad, Pakistan; ^7^Institute of Pharmaceutical Sciences, University of Veterinary and Animal Sciences, Lahore, Pakistan; ^8^Department of Pharmacy, Quid-i-Azam University, Islamabad, Pakistan

**Keywords:** awareness, public, COVID-19, pakistan, knowledge, practices

## Abstract

**Objectives:** Effective mitigation of coronavirus diseases (COVID-19) pandemic requires true adoption of precautionary measures by the masses, that primarily depends upon their knowledge and practices behaviors. The current study aimed to assess the knowledge; practices of Pakistani residents regarding COVID-19 and factors associated with adequate knowledge and positive practices.

**Material and Methods:** A cross-sectional online survey was conducted from 15-April 2020 to 20 May 2020 among 689 Pakistanis by using a validated self-administered questionnaire (Cronbach's alpha 0.77). The questionnaire included questions on the assessment of demographics, the source of information, knowledge, and practice of COVID-19 on google forms and shared links with the WhatsApp groups, Facebook pages and other online platforms. Regression analysis was applied to find potential predictors of knowledge and practices.

**Results:** Of 689 participants, 431 (62.6%) were male, 64.3% (*n* = 443) were aged <30 years, and\328 (47.6%) of participants were married. 48.19% (*n* = 332) had adequate knowledge; 81% (*n* = 555) had positive practices regarding COVID-19 and majority (66.62%, *n* = 459) seek knowledge from social media. Knowledge was significantly higher (OR > 1.00, *p* < 0.05) among educated and higher income participants. Positive practices were significantly (OR > 1.00, *p* < 0.05) related to the older age (≥50 years), higher education, higher income and good knowledge regarding COVID-19.

**Conclusion:** The study concluded that Pakistani residents had average knowledge and good practices toward COVID-19 yet there are gaps in specific aspects of knowledge, and practice that should be focused in future awareness and educational campaigns. The study recommends the ministry of health authorities to promote all precautionary and preventive measures of COVID-19-consisting of a better-organized approach to all strata of society: less privileged people, older ones and less educated people, in order to have equilibrium of knowledge about COVID-19; hence effective implementation of precautionary measures.

## Introduction

On 11 March 2020, the World Health Organization (WHO) declared the outbreak of coronavirus disease (COVID-19) to be a pandemic, with disease spread in 114 countries and more than 4,000 deaths (1). On 31 May 2020, there were 6,175,290 cases of COVID-19, with 371,228 confirmed deaths in 213 countries and territories worldwide (2). Pakistan, a lower middle-income country (LMIC), ranked 5th among the most populous countries in the world to be considered the new COVID-19 hotspot with a fragmented health care system (3). As far as COVID-19 is concerned, Pakistan has a uniquely challenging situation due to its vulnerable geographical location, as it shares borders with China and Iran due to poor screening capacity, leading to delayed implementation of preventive measures (4). At the beginning of January 2020, the WHO sent technical assistance to Pakistan, at which time Pakistan did not have a COVID-19 case or even a COVID-19 test capability. WHO and Pakistan's Ministry of Health have begun to investigate possible cases and to distribute disease risk information brochures to passengers arriving initially at airports (5). Pakistan's first case of COVID-19 was reported on 26 February in Karachi, having a travel history of Iran (5). Within 6 weeks, Pakistan developed a test capacity of up to 30,000 tests per day, imposed lockdowns across the country, suspending public transportation systems, and restricting air and sea travel (5). Due to the lockdown and limited follow-up to standard operating procedures (SOPs), the country's healthcare systems have been burdened by rapidly increasing cases (6). Pakistani Prime Minister Imran Khan has taken the initiative and launched the “Smart lock-down” campaign to limit unnecessary movements in cities (6). Pakistan has also set up a National Command Operations Center (NCOC), which combines military and government to produce and distribute cheap masks locally and developed the guidelines to curb COVID-19 (6). However, as of 31 May 2020, Pakistan has 68,270 laboratory-confirmed cases and 1,483 COVID-19 associated deaths. Sindh (*n* = 27,360) has the highest number of cases followed by Punjab (*n* = 25,056), Khyber Pakhtunkhwa (KP) (*n* = 9,540), Baluchistan (*n* = 4,193), Islamabad (*n* = 2,418), Gilgit Baltistan (678), and Azad Jammu Kashmir (*n* = 251) (7). Punjab (*n* = 475) being the most populous province has the highest number of deaths followed by Sindh (*n* = 465), KP (*n* = 453), Baluchistan (*n* = 46), Islamabad (*n* = 27), Gilgit Baltistan (10), and Azad Jammu Kashmir (*n* = 7) (7).

People may not have access to regular and reliable sources of disease etiology information in the context of LMICs, leaving them ill-equipped to minimize the risk of infection in emerging outbreaks (8–10). Concerns have arisen about the possibility of obstructing public health communication due to misinformation (11–13). As WHO Director-General Dr. Tedros Adhanom Ghebreyesus said, “we're not just fighting an epidemic; we're fighting an infodemic” (14). Excessive or understated pandemic estimates may either give the public a fuel panic or a falsified sense of security (15). Confusion of basic information about how the virus can be reduced and how it can be exposed puts people at risk of infection (16). Data suggest that knowledge, awareness, perception, and attitude of the general public regarding disease play an essential role in the control and management of disease as observed in epidemics of SARS and MERS (14). Poor knowledge of the general public regarding preventive measures as avoiding crowded areas, wearing masks, properly washing hands, and maintaining social distance also pose a significant gap in control of the spread of COVID 19 (17). High number of people with a lack of symptoms could be a possible way of the virus spread (18). Deeming public awareness to be crucial in preventing the spread of COVID−19, which otherwise lacks effective treatment and preventive measures, vast public awareness campaigns are critical in the fight against it (19).

The Pakistani National Institute of Health (NIH) has played a vital role in designing and circulating protocols regarding COVID-19 transmission and prevention, as well as launching public awareness campaigns (7). However, final success depends upon the adherence of people to guidelines and preventive measures that are strongly linked to their understanding and awareness toward disease (20). Survey highlighting awareness level is useful to get information regarding public health education, response and recovery efforts, and social mobilization (21). Data from the study is pivotal for policy development and public health implementation to respond to the outbreak shortly and consistently quickly. In context of the explanation above, the current study aimed to evaluate the current level of awareness regarding transmission, symptoms, and preventive measures of COVID-19 among the general population in Pakistan. Additionally, this study will provide a snapshot of the extent of precautionary measures practiced by the Pakistani population.

## Methods

### Study Design

A cross-sectional survey-based study was conducted during the months of April and May 2020, days of strict lockdown to implement social distancing to avoid the spread of the pandemic. The investigators opted for an online data collection method because it was not possible to carry out a population-based survey in this critical/censorious situation.

### Sampling, Study Population and Data Collection Method

The sample size calculated by Raosoft was 583, assuming a response rate of 50%, confidence interval (CI) 95%, Z as 1.96, and margin of error d as 4%. Considering, an additional 20% (*n* = 116) for any error in questionnaire filling, a final sample size of 699 will be required. The survey was started on 15 April 2020, and response acceptance was closed on 20-May 2020, when required sample size was achieved. Pakistani people 16 years of age or older who voluntarily agreed to fill out the form/questionnaire were selected. Participants were given no monetary benefits to participate in the study due to lack of funds.

The questionnaire was designed on google forms and the generated link was shared with the WhatsApp groups. Link was also shared personally with the contact list of investigators.

### Measure

A survey instrument was designed based on substantial literature analysis (22–24), material related to emerging respiratory diseases including COVID-19 by WHO (25) and guidelines issued by national institute of health (NIH), Islamabad Pakistan (7). After the preliminary draft questionnaire was drawn up, it was validated in two stages. In the first place, the study tool was discussed with pharmacy and medical researchers and professionals to give their expert opinion on its simplicity, relativity and relevance. Secondly, a pilot study was conducted by selecting a small sample (*n* = 60) to make the questionnaire simpler and more comprehensive. The questionnaire was amended based on the suggestions made by the participants and its consistency with the published literature. After a thorough discussion, the authors finalized the questionnaire and then distributed it to the participants for their response. The coefficient of reliability was calculated using SPSS v.20 and Cronbach's alpha value. It was found to be 0.77. The data from the pilot study were not included in the final analysis.

The questionnaire included questions on the assessment of demographics, the source of information, knowledge, and practice of COVID-19. The demographic characteristics included gender, age, marital status, monthly income, residence, employment status and education. One item was regarding the source of information about COVID-19. Awareness section comprised of 20 items; regarding etiology (2-items), symptoms (7-items), risk group (1-item), transmission (6-items), treatment (2-items) and precautions/preventions (2-items). Each question was responded as Yes, No, and I don't know. The correct answer was marked as 1 while wrong answer was marked as 0. Total score ranges from 0 to 14, and a cut off level of ≤15 was set for poor knowledge and ≥16 (More than 75%) for good knowledge.

The practice section included 6 items related to the use of face mask, and implementation of other precautionary measures were included in the practice section. Each item was responded as yes (1-point), No (0-point), and sometimes (0-point). Practice items total score ranged as 0-6, where 5–6 score was considered as good practice, and a score of 1–4 indicated poor practice of preventive measures for COVID-19.

### Ethics

The study was performed following the declaration of Helsinki. Due to lockdown, Universities were closed, hence study protocol was approved from the “Tehsil headquarter hospital samundri” hospital board (767/THQ/HR). The study questionnaire contained a consent portion that stated purpose, nature of the survey, study objectives, volunteer participation, declaration of confidentiality, and anonymity.

### Statistical Analysis

Data were entered in Microsoft Excel and later imported into SPSS V.21 for statistical analysis. Numerical variables were measured as mean and standard deviations, while categorical variables were expressed as frequencies and percentages. Inferential statistics were applied depending upon the nature of data and variables. Chi-square tests were used to find differences in knowledge groups (good vs. poor) and practice (good vs. poor) by demographic characteristics. Binary logistic regression models have been used to identify possible determinants of good knowledge and practice, both unadjusted and adjusted (adjusted for age, gender and other demographic variables). Results were expressed as crude odds ratio (COR), and adjusted odds ratio (AOR) accompanied by 95% confidence interval (CI). A *p*-value of < 0.05 will be considered significant in all tests.

## Results

Out of the total 699 responses collected, 10 questionnaires were excluded due to missing information, and 689 responses were analyzed in the final analysis. Most participants were male (62.6%, *n* = 431), 64.3% (*n* = 443) were aged < 30 years, and less than half (47.6%, *n* = 328) of participants were married. More than half (53.3%, *n* = 367) of respondent had monthly income of (Pakistani rupee) PKR 0 - 24,999, 76.8% (*n* = 529) were unemployed, and 65% (*n* = 448) participants had education of 13 years or more (Table 1).

**Table 1:** Demographic characteristics of study population (*N* = 689).

**Variables**	**Characteristics**	**Number of participants (n)**	**Percentage (%)**
**Gender**			
	Female	258	37.4
	Male	431	62.6
**Age**			
	< 30 years	443	64.3
	31–39 Years	148	21.5
	40–49 years	69	10.0
	More than 50 years	29	4.2
**Marital status**			
	Single	361	52.4
	Married	328	47.6
**Monthly Income**			
	0–24,999	367	53.3
	25, 000–49,999	165	23.9
	≥50,000,	157	22.8
**Employment status**			
	Employed	160	23.2
	Unemployed	529	76.8
**Residence**			
	Rural	160	23.2
	Urban	529	76.8
**Education status**			
	None	71	10.3
	≤ 10 years	39	5.7
	11–12	131	19.0
	13 or more	448	65.0

**Figure 1:**
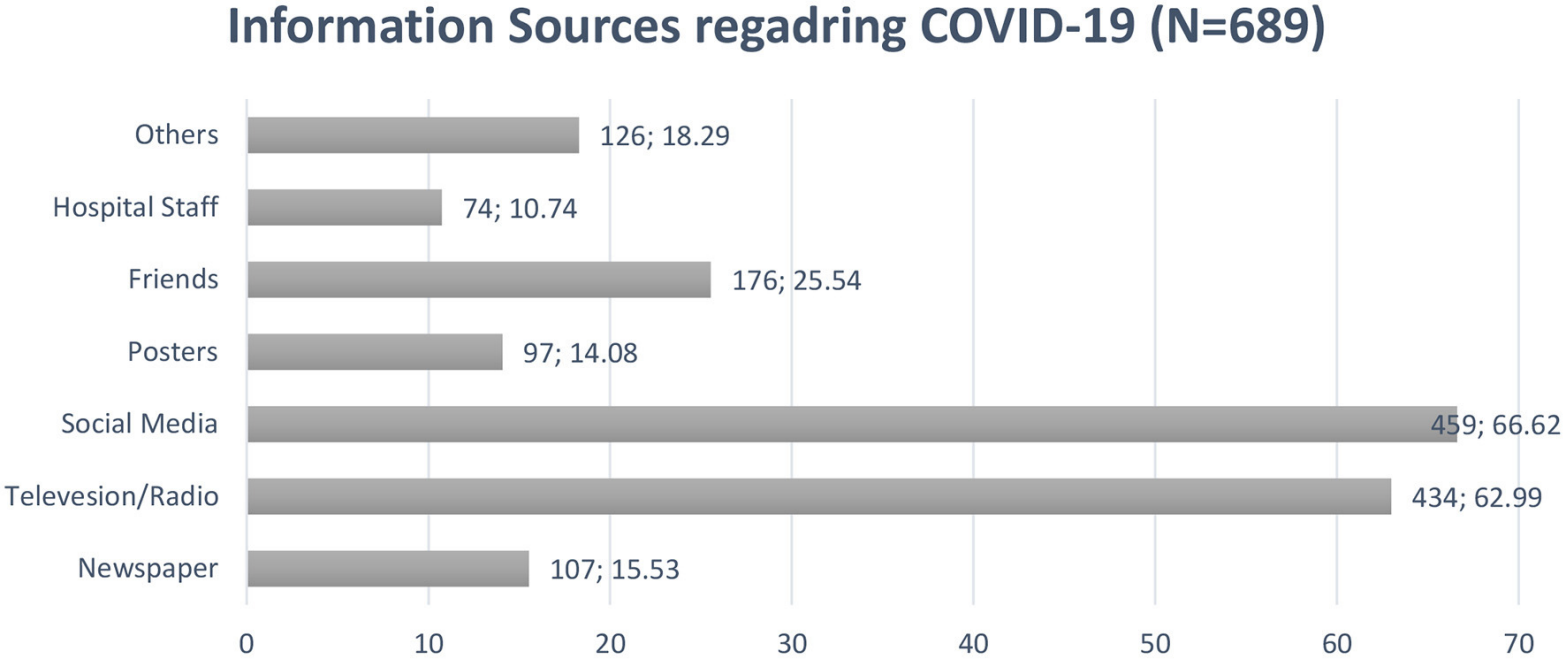
Information sources utilized by public in Pakistan.

Figure 1 provides a summary of the information sources utilized by respondents. Majority of participants used social media (66.62%, *n* = 459) as a source to seek information regarding COVID-19 followed by television/radio (62.99%, *n* = 434) and friends (25.54%, *n* = 176).

### Knowledge Regarding COVID-19

**Figure 2:**
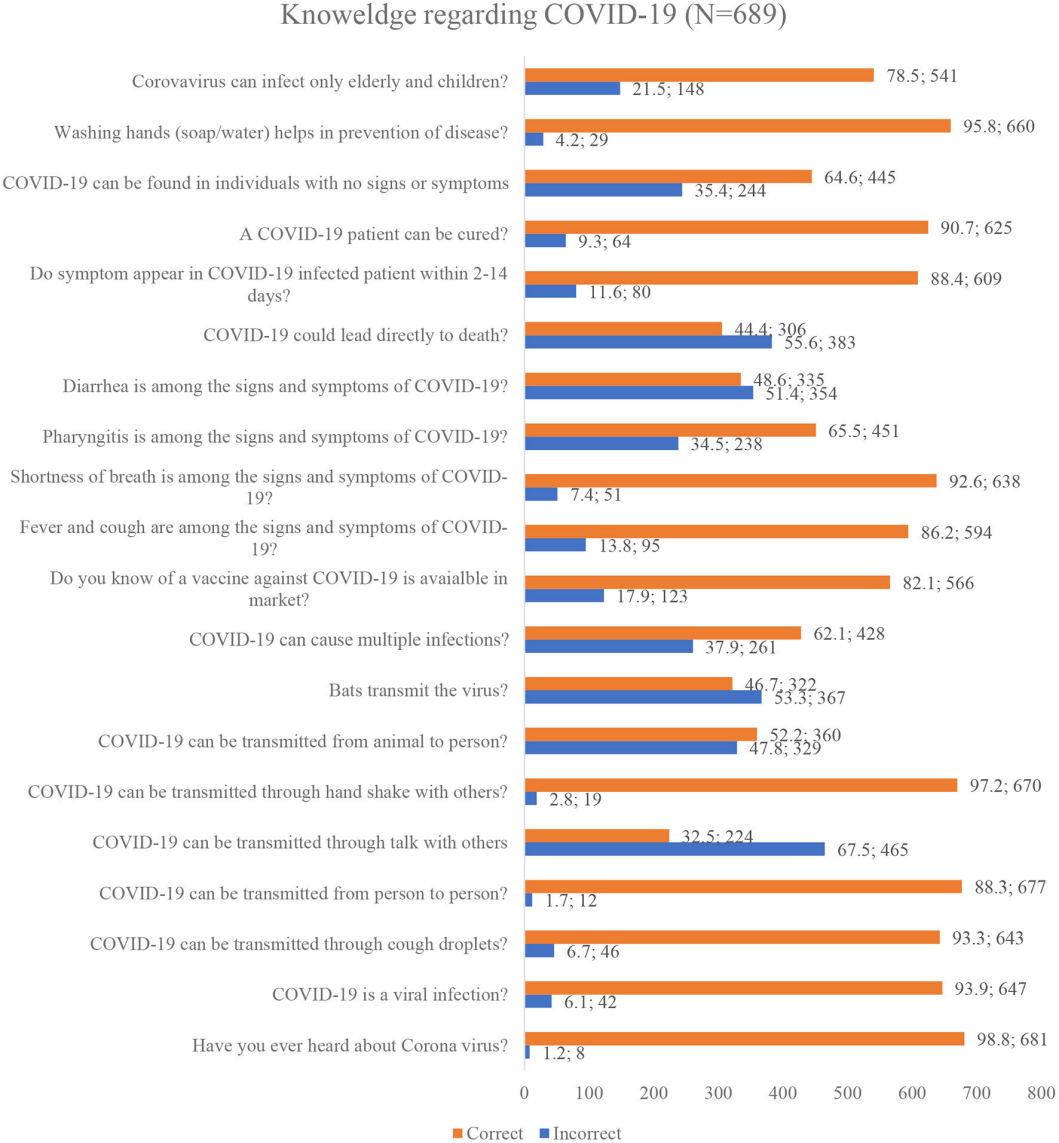
Public's Knowledge regarding COVID-19.

Figure 2 summarizes the responses of participants for knowledge items of the questionnaire. Mixed responses were obtained regarding 20 items. The majority of the 689 participants had heard about the disease, and 93.9 % (*n* = 647) know that virus is the causative agent of COVID-19. Response to questions regarding transmission of disease indicated 93.3% (*n* = 643) participants correctly identified that virus can transmit trough droplets, 97.2% (*n* = 670) subjects were well aware that infection can be transmitted by shaking hands, and 98.3% (*n* = 677) respondents had correct knowledge regarding the transmission of the virus from person to person. On the other hand, when the question asked regarding fatality of disease, 55.6% (*n* = 383) subjects respond that COVID-19 directly leads to death, 35.4% (*n* = 244) respondent did not know that COVID-19 can be found in a person with no signs and symptoms, and 21.5% (*n* = 148) reported that virus could affect only elderly and children.

### Practices Regarding COVID-19

**Figure 3:**
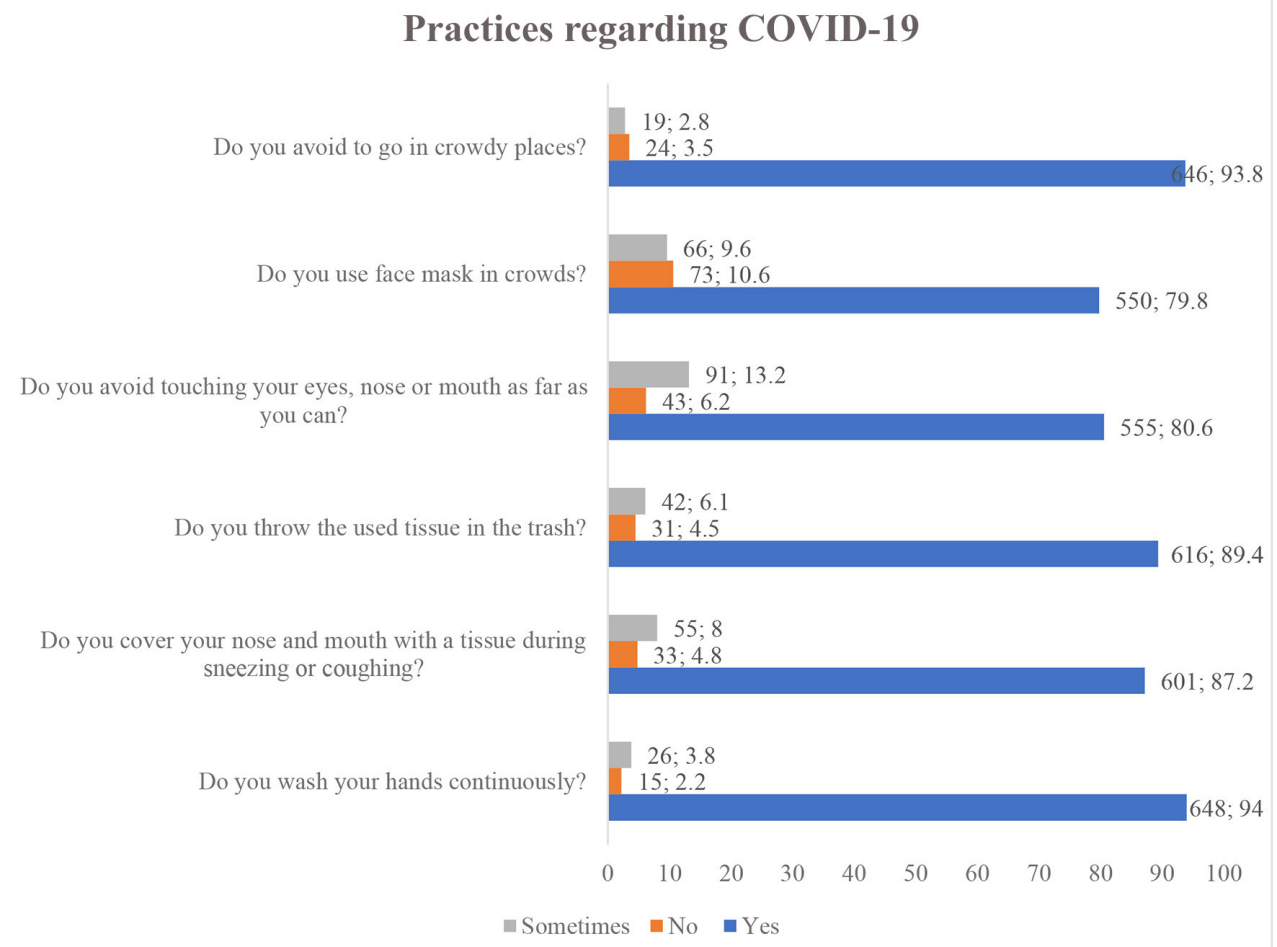
Public practices regarding COVID-19.

Figure 3 summarizes the practices of the general population regarding COVID-19. All of the 689 participants respond to all 6 items regarding COVID-19. Most respondents had good practice regarding each item with the highest practice showed toward washing of hands with soap or cleaning with sanitizers (94%, *n* = 648), and a similar proportion (93.8%, *n* = 646) of participants revealed that they avoid going in a crowded place. A lower percentage of good practice was observed among the general population to wear a face mask (79.8%, *n* = 550).

### Difference in Knowledge and Practice Status by Demographics

More than half of participants had poor knowledge (51.81%, *n* = 357), while 332 (48.19%) individuals had adequate knowledge regarding COVID-19. Chi-square tests were applied to find differences in knowledge status by sample characteristics. Knowledge status was significantly differed by monthly income as participants with higher income have adequate knowledge compared to lower-income counterparts (χ^2^ = 25.85, *p* < 0.001). The findings showed that knowledge status differed significantly from marital status (χ^2^ = 4.606, *p* = 0.032), to employment status (χ^2^ = 4.968, *p* = 0.026). Similarly, most participants with higher education had adequate knowledge compared to less-educated counterparts (χ^2^ = 24.07, *p* < 0.001) while the status of practice did not differ significantly in terms of gender, age, and residence (*p* > 0.05) (Table 2).

**Table 2:** Difference in knowledge and practice status by demographics.

**Characteristics**	**Categories**	**Knowledge**		**χ^2^ (P)**	**Practice**		**χ2 (P)**
		**Poor knowledge**	**Good knowledge**		**Poor practice**	**Good practice**	
		***n* (%)**	***n* (%)**		***n* (%)**	***n* (%)**	
Total		357 (51.81)	332 (48.19)		134 (19)	555 (81)	
Gender				0.273 (0.601)			0.690 (0.406)
	Female	137 (53.1)	121 (46.9)		46 (17.8)	212 (82.2)	
	Male	220 (51.0)	211 (49.0)		88 (20.4)	343 (79.6)	
Age				5.020 (0.170)			7.011 (0.071)
	<30 years	217 (49.0)	226 (51.0)		83 (18.7)	360 (81.3)	
	31–39 Years	88 (59.5)	60 (40.5)		20 (19.6)	119 (80.4)	
	40–49 years	36 (52.2)	33 (47.8)		11 (15.9)	58 (84.1)	
	More than 50 years	16 (55.2)	13 (44.8)		11 (37.9)	18 (62.1)	
Marital status				4.606 (0.032)			0.534 (0.465)
	Single	173 (47.9)	188 (52.1)		74 (20.5)	287 (79.5)	
	Married	184 (56.1)	144 (43.9)		60 (18.3)	268 (81.7)	
Monthly Income				25.85 (<0.001)			8.979 (0.011)
	0–24,999	223 (60.8)	144 (39.2)		85 (23.2)	282 (76.8)	
	25, 000–49,999	65 (39.4)	100 (60.6)		20 (12.1)	145 (87.9)	
	≥50,000,	69 (43.9)	88 (56.1)		29 (18.5)	128 (81.5)	
Employment status				4.968 (0.026)			2.554 (0.110)
	Employed	215 (48.6)	227 (51.4)		78 (17.6)	364 (82.4)	
	Unemployed	142 (57.5)	105 (42.5)		56 (22.7)	191 (77.3)	
Residence				0.784 (0.376)			6.154 (0.013)
	Rural	78 (48.8)	82 (51.2)		42 (26.3)	118 (73.8)	
	Urban	279 (52.7)	250 (47.3)		92 (17.4)	437 (82.6)	
Education status				24.007 (<0.001)			16.716 (0.001)
	None	44 (62.0)	27 (38.0)		16 (22.5)	55 (77.5)	
	≤ 10 years	32 (82.1)	7 (17.9)		17 (43.6)	22 (56.4)	
	11–12	74 (56.5)	57 (43.5)		25 (19.1)	106 (80.9)	
	13 or more	207 (46.2)	241 (53.8)		76 (17.0)	372 (83.0)	

Findings indicated that 81% (*n* = 555) participants had good practice in following precautionary measures regarding COVID-19. Chi-square analysis revealed that participant's practices regarding COVID-19 were significantly differed by monthly-income (χ^2^ = 8.979, *p* = 0.011), residence (χ^2^ = 6.154, *p* = 0.013), and education status (χ^2^ = 16.716, *p* = 0.001). While the status of the practice did not differ significantly in terms of gender, age, marital status, and employment status (*p* > 0.05) (Table 2).

### Binary Logistic Regression Analysis for Factors Associated With Good Knowledge and Practice

Adjusted and un-adjusted regression analysis was applied to find possible predictors of good knowledge and practice among the general population in Pakistan. The regression model adjusted for all independent variables showed that participants with higher monthly income (≥50,000) had higher odds (AOR: 2.133, 95% CI: 1.394–3.867, *p* < 0.001) of adequate knowledge compared to the reference category. Similarly, participants who have an education of 13 years or more had higher odds (AOR: 1.501, 95% CI: 0.858–2.627, *p* = 0.041) of good knowledge compared to less-educated counterparts (Table 3).

**Table 3:** Binary logistic regression analysis for factors associated with good knowledge.

**Characteristics**	**Factors associated with good knowledge**						
		**COR**	**95% CI**	**P**	**AOR**	**95% CI**	**P**
**Gender**							
	Female	Reference	–	–	Reference	–	–
	Male	1.086	0.797–1.479	0.601	1.153	0.819–1.624	0.415
**Age**							
	<30 years	Reference	–	–	Reference	–	–
	31–39 Years	0.655	0.449– 0.955	0.028	0.642	0.393–1.050	0.078
	40–49 years	0.880	0.530– 1.462	0.622	0.831	0.441–1.567	0.567
	More than 50 years	0.965	0.367– 1.660	0.519	0.859	0.358–2.062	0.859
**Marital status**							
	Single	Reference	–	–	Reference	–	–
	Married	0.720	0.533–0.972	0.032	0.742	0.487–1.131	0.742
**Monthly Income**							
	0–24,999	Reference	–	–	Reference	–	–
	25, 000–49,999	2.382	1.636–3.470	<0.001	2.420	1.576–3.717	<0.001
	≥50,000,	2.975	1.753–3.884	<0.001	2.133	1.394–3.867	<0.001
**Employment status**							
	Employed	Reference	–	–	Reference	–	–
	Unemployed	0.700	0.512–0.958	0.026	0.819	0.566–1.186	0.291
**Residence**							
	Rural	Reference	–	–	Reference	–	–
	Urban	0.852	0.598–1.214	0.376	0.704	0.480–1.035	0.704
**Education status**							
	None	Reference	–	–	Reference	–	–
	≤ 10 years	0.356	0.138–0.920	0.033	0.348	0.130–0.931	0.035
	11–12	1.255	0.695–2.286	0.451	1.086	0.580–2.035	0.797
	≥13	1.897	1.135–3.172	0.015	1.501	0.858–2.627	0.041

Finding showed that age group of 50 years or more (AOR: 1.087, 95% CI: 0.428–1.207, *p* = 0.020), monthly income of PKR 25, 000 - 49,999 (AOR: 1.875, 95% CI: 1.055–3.331, *p* = 0.032), education of 11–12 years (AOR: 1.18, 95% CI: 0.567–2.489, *p* = 0.049), education of 13 years or more (AOR: 1.250, 95% CI: 0.647–2.415, *p* = 0.031), and good knowledge (vs. Poor knowledge: AOR: 1.80, 95% CI: 1.201–2.700, *p* = 0.004) were the substantial determinants of good practice regarding COVID-19 among general population in Pakistan (Table 4).

**Table 4:** Binary logistic regression analysis for factors associated with good practice.

**Characteristics**	**Factors associated with good practice**						
		**COR**	**95% CI**	**P**	**AOR**	**95% CI**	**P**
**Gender**							
	Female	Reference	–	–	Reference	–	–
	Male	0.846	0.569–1.256	0.406	0.810	0.528–1.1242	0.334
**Age**							
	<30 years	Reference	–	–	Reference	–	–
	31–39 Years	0.946	0.591–1.515	0.818	0.740	0.396–1.381	0.344
	40–49 years	1.121	0.611–2.417	0.578	0.920	0.394–2.145	0.847
	More than 50 years	1.377	1.072–1.829	**0.015**	1.087	0.428–1.207	**0.020**
**Marital status**							
	Single	Reference	–	–	Reference	–	–
	Married	1.152	0.788–1.682	0.465	1.445	0.834–2.503	0.189
**Monthly Income**							
	0–24,999	Reference	–	–	Reference	–	–
	25, 000–49,999	2.185	1.291–3.700	0.004	1.875	1.055–3.331	**0.032**
	≥50,000,	2.330	1.831–3.130	0.234	1.962	1.251–2.120	0.379
**Employment status**							
	Employed	Reference	–	–	Reference	–	–
	Unemployed	0.731	0.497–1.074	**0.111**	0.891	0.573–1.386	0.609
**Residence**							
	Rural	Reference	–	–	Reference	–	–
	Urban	1.691	1.113–2.568	0.014	1.363	0.876–2.120	0.170
**Education status**							
	None	Reference	–	–	Reference	–	–
	≤ 10 years	0.376	0.162–0.875	0.561	0.415	0.173–0.995	0.649
	11–12	1.233	0.608–2.501	**0.023**	1.188	0.567–2.489	**0.049**
	13 or more	1.424	0.775–2.618	**0.015**	1.250	0.647–2.415	**0.031**
**Knowledge**							
	Poor	Reference	–	–	Reference	–	–
	Good	1.878	1.271–2.774	**0.002**	1.801	1.201– 2.700	**0.004**

*Bold values shown significant factors*.

## Discussion

In view of the rapid spread of COVID-19 and the increase in the number of cases in Pakistan, it is necessary to have a clear picture of the state of public awareness and their practices in the context of the precautionary measures. In addition, Pakistan is a populous country and is facing enormous pressure on non-communicable diseases (26). Both factors increase the country's vulnerability to this deadly infection and results in higher mortality and morbidity. Moreover, Pakistan's history of dealing with epidemics required a high level of preparedness by government as well as masses (27). Global efforts have been made to reduce the transmission of this contagious infection. These efforts include political efforts by the governments, together with personal attitudes and behaviors, which depend on the awareness of the public about the disease.

Findings revealed that almost half of the population had good knowledge, and 80% had a precautionary approach. News channels such as the internet and social media platforms had become commonly used information sources compared to traditional channels such as newspapers etc. Social media was the primary source of information to be used by the public (66.62%) in Pakistan to obtain information on COVID-19. This could be explained by the fact that 64.3% of study participants were under 30 years of age and had University level education. This stratum is the main user of the Internet in Pakistan, according to a recent survey by the Pakistan Telecommunications Authority (PTA), 63% of the 76 million people who have access to the Internet are under 30 years of age (28).

This finding has implications that although social media platforms could be an easily accessible source of information, there is a potential risk of misinformation (13, 29). As with this pandemic of COVID-19, there is also a pandemic of misinformation on the Internet that leads to negative reactions from the public (13). Mainly, false information regarding the potential benefits of certain drugs such as hydroxychloroquine stimulated the irrational use of this drug by masses, and this results in a shortage of these medicines and becomes unavailable to patients who need (13).

Results show that 48.19% (*n* = 332) of the population had adequate knowledge of the nature, transmission, risk groups and precautionary measures of COVID-19. This rate of adequate knowledge is lower as reported in a Pakistani study (64.8%) (30), a Malaysian study (80.5%) (31) and a Chinese study (90%) (23) while higher than a Ethiopian study (36.7%) (12). However, this is in agreement with the findings of Abdelhafiz et al., who reported Egyptians had average (16.39 ± 2.63, range: 7–22) knowledge regarding COVID-19 (22). A possible reason for less knowledge reported in this study could be explained by the fact that most respondents attained COVID-19 related information from social media. Owing to unauthenticated and the use of social media to get information can explain the existence of myths and misinformation among the public (32).

Of note that more than half (55.6%, *n* = 383) of the study population incorrectly reported that COVID-19 directly leads to death. While wolf et al. reported that only 14.2% of US adults think that COVID-19 may cause death (24). Possible speculation is that factors such as the outbreak itself and consequential lockdown result in severe psychological impact as khan et al. reported 87.73% of the studied population feared the current situation, which leads to fatigue, anxiety, and depression (33). Additionally, misinformation surging on the internet and related economic pressure also put immense pressure and creates negative feelings about the situation (34).

The adjusted regression model revealed that higher education and monthly income are substantial predictors of good knowledge (*P* < 0.05). These results are in line with an Egyptian study which also stated that public awareness was important in terms of its socioeconomic status and level of education (*P* < 0.002) (22). A Chinese study concluded that higher education (middle school or lower vs. Master β: 1.346, *P* < 0.001) played a significant role in increasing good knowledge (23). While an American study didn't found any difference in respondents' knowledge regarding symptoms by poverty level (68.5% vs. 73.1%, *P* > 0.05) (24), this finding is of particular importance for the Government and the authorities concerned to focus on the less privileged stratum of society to ensure the effective implementation of precautionary measures.

Findings indicated that 80% of participants had positive practices in following precautionary measures. This is in line with the results of Zhong et al., and Alahdal et al., who also reported that the majority (>90%) and (81%) of participants were following precautionary measures (23, 35). Possible speculation of a higher rate of good practices depsite of only 50% population had good knowledge could be that of campaigns lauched by Government describing causes, symptoms, and route but these awareness campaigns primarily focused on highlighting precautionary measures such as wearing a facemask, social distancing, and hand hygiene practices.

Note that 20.2% of the participants did not wear a face mask when they left their homes. This poroprtion is much higher than the chineese study, which reported that only 2% of the population studied did not wear a face mask (23). While Pakistani study reported that 14.2% of the people surveyed did not wear a face mask. Despite the vigorous broadcast of precautionary measures, this risk of taking action could be attributed to the younger age of the participants. Additionally, this might be because face mask shortage in different parts of the country due to high demand as well as price hiking also affects the affordability of the less income stratum. The government had taken several measures to ensure the availability and price control of all personal protective equipment (PPEs).

The adjusted multivariable logistic regression model demonstrated that older age, higher education, and knowledge regarding COVID-19 are the factors that substantially related to the positive attitude among the public in Pakistan. Zhong et al. found that knowledge was significantly associated with positive practices as Chinese individuals with higher education regarding COVID-19 were less likely to visit crowded places (OR:0.90, *p* = 0.001) and not wearing a face mask (OR:0.78, *p* < 0.001) (23). While a Pakistani study also concluded that younger age (vs. 30 years; OR = 3.08, *p* < 0.001) and lower education (matriculation vs. Master degree, OR = 6.829, *p* < 0.001) were the characteristics potentially associated with poor practices regarding COVID-19 (30).

To control the pandemic, there is a need for continuous monitoring of the implementation of preventive measures, the review of existing interventions and the updating of such responses. This study helps to inform the current state of awareness and practices of preventive measures and adds to the findings of a previously conducted study in Pakistan (30).

Regarding the policy implications, the findings will reconsider the involvement of the community as a key approach to the fight against any outbreak, including COVID 19. Generally, the current survey data most likely showed that the government's recommendation for the desired preventive action is positive for public health education.

## Limitations

There are several implicit limitations to the study. First, as this is an online survey design, the response depends primarily on honesty and partly on the ability to recall and may, therefore, be subject to bias recall. However, due to a policy of lockdown and social distancing, the survey hand filling was not possible. Second, the sample size is not large enough, and most of the respondents were from one province (Punjab), which limits the generalizability of the population in the whole Pakistan. An additional limitation of the study is that most of the respondents belong to a population age group least affected by the pandemic (i.e., younger adults), while there is a lower representation of the age group most affected (i.e., older adults).

## Conclusion

This quick online survey shows that around half of Pakistani residents had keen awareness, and 81% had positive practices following precautionary steps. The study is also capable of highlighting gaps in specific aspects of knowledge and practice that should be addressed in future awareness and education campaigns. Findings have also shown that fewer credible sources are used by the general population, which should be discussed immediately because it eventually affects information and is demonstrated in attitudes and practices. The study suggests that the Ministry of Health supports both the corrective and therapeutic steps of COVID-19, consisting of a better-organized approach to all strata of society, i.e., the less privileged, the elderly and the less educated, to provide a balance of knowledge of COVID-19 and hence successful implementation of the precautionary measures.

## Impacts

- Pakistani residents had average knowledge and good practices of following precautionary measures for COVID-19.
- Findings have shown that education and economic status are potential predictors of good knowledge and positive practices.
- The study findings require future interventions to be targeted at all strata of society: the less privileged, the elderly and the less educated, to balance knowledge toward COVID-19.

## Data Availability Statement

The raw data supporting the conclusions of this article will be made available by the authors, without undue reservation.

## Ethics Statement

The studies involving human participants were reviewed and approved by from the Samundari hospital board (767/THQ/HR). Written informed consent to participate in this study was provided by the participants' legal guardian/next of kin.

## Author Contributions

The manuscript idea, concept, writing, and layout was done by MS, AA, and SN. IN, AG, and MM provided critical help in writing, statistical and layout designing. IN, AA, and ZA provided critical input regarding data analysis at every step of the manuscript writing process. MS, SK, and ZA proofread the manuscript and provided input in formulating the draft. All authors contributed to the article and approved the submitted version.

## Conflict of Interest

The authors declare that the research was conducted in the absence of any commercial or financial relationships that could be construed as a potential conflict of interest.

## References

[1] Organization WH. WHO Director-General's Opening Remarks at the Media Briefing on COVID 19. Genebra: World Health Organization (2020).

[2] Worldometer. Coronavirus Cases. (2020). Available online at: https://www.worldometers.info/coronavirus/ (accessed May 31, 2021).

[3] Slater J Masih N. Home to Nearly 2 Billion People, South Asia Could be the Next Coronavirus Hot Spot. (2020). Available online at: https://www.washingtonpost.com/world/asia_pacific/home-to-nearly-2-billion-people-south-asia-could-be-the-next-coronavirus-hot-spot/2020/03/19/35431fbe-6918-11ea-b199-3a9799c54512_story.html (accessed May 31, 2020).

[4] Saqlain M Munir MM Ahmed A Tahir AH Kamran S. Is Pakistan prepared to tackle the coronavirus epidemic? Drugs Ther Pers. (2020) 36:213–4. 10.1007/s40267-020-00721-1 32218652 PMC7095264

[5] WHO. COVID-19 in Pakistan: WHO Fighting Tirelessly Against the Odds. (2020). Available online at: https://www.who.int/news-room/feature-stories/detail/covid-19-in-pakistan-who-fighting-tirelessly-against-the-odds (accessed January 01, 2021).

[6] Ashraf I. Pakistan's Strategies to Face COVID-19's Destruction. Islamabad: Daily Sabah (2020).

[7] Pakistan N. COVID-19 Health Advisory Platform by Ministry of National Health Services Regulations and Coordination. (2020). Available online at: http://covid.gov.pk/ (accessed May 31, 2020).

[8] Reuben RC Danladi MM Saleh DA Ejembi PE. Knowledge, attitudes and practices towards COVID-19: an epidemiological survey in north-central Nigeria. J Comm Health. (2020). 10.1007/s10900-020-00881-1. [Epub ahead of print]. 32638198 PMC7338341

[9] Ferdous MZ Islam MS Sikder MT Mosaddek ASM Zegarra-Valdivia J Gozal D. Knowledge, attitude, and practice regarding COVID-19 outbreak in Bangladesh: an online-based cross-sectional study. PLoS ONE. (2020) 15:e0239254. 10.1371/journal.pone.0239254 33035219 PMC7546509

[10] Lee H Moon SJ Ndombi GO Kim K-N Berhe H Nam EW. COVID-19 perception, knowledge, and preventive practice: comparison between South Korea, ethiopia, and democratic republic of congo. Afr J Rep Health. (2020) 24:66–77. 10.29063/ajrh2020/v24i2s.11 34077056

[11] Bedford J Enria D Giesecke J Heymann DL Ihekweazu C Kobinger G. COVID-19: towards controlling of a pandemic. Lancet. (2020) 395:1015–8. 10.1016/S0140-6736(20)30673-5 32197103 PMC7270596

[12] Desalegn Z Deyessa N Teka B Shiferaw W Hailemariam D Addissie A. COVID-19 and the public response: knowledge, attitude and practice of the public in mitigating the pandemic in addis ababa, ethiopia. PLoS ONE. (2021) 16:e0244780. 10.1371/journal.pone.0244780 33411766 PMC7790293

[13] Malik M Tahir MJ Jabbar R Ahmed A Hussain R. Self-medication during Covid-19 pandemic: challenges and opportunities. Drugs Ther Pers. (2020) 36:565–7. 10.1007/s40267-020-00785-z 33041621 PMC7532737

[14] Lau LL Hung N Go DJ Ferma J Choi M Dodd W. Knowledge, attitudes and practices of COVID-19 among income-poor households in the philippines: a cross-sectional study. J Glob Health. (2020) 10:011007. 10.7189/jogh.10.011007 32566169 PMC7294392

[15] Dardas LA Khalaf I Nabolsi M Nassar O Halasa S. Developing an understanding of adolescents' knowledge, attitudes, and practices toward COVID-19. J School Nurs. (2020) 36:430–41. 10.1177/1059840520957069 32990150

[16] Narayana G Pradeepkumar B Ramaiah JD Jayasree T Yadav DL Kumar BK. Knowledge, perception, and practices towards COVID-19 pandemic among general public of India: a cross-sectional online survey. Curr Med Res practice. (2020) 10:153–9. 10.1016/j.cmrp.2020.07.013 32839725 PMC7372279

[17] Khan Z Muhammad K Ahmed A Rahman H. Coronavirus outbreaks: prevention and management recommendations. Drugs The Pers. (2020) 36:215–7. 10.1007/s40267-020-00717-x PMC7095077 32218651

[18] Anderson RM Heesterbeek H Klinkenberg D Hollingsworth TD. How will country-based mitigation measures influence the course of the COVID-19 epidemic? Lancet. (2020) 395:931–4. 10.1016/S0140-6736(20)30567-5 32164834 PMC7158572

[19] Kuang J Ashraf S Das U Bicchieri C. Awareness, risk perception, and stress during the COVID-19 pandemic in communities of Tamil Nadu, India. Int J Environ Res Public Health. (2020) 17:7177. 10.3390/ijerph17197177 PMC7579253 33007992

[20] Ahmed A Tanveer M Saqlain M Khan GM. Knowledge, perception and attitude about crimean congo hemorrhagic fever (CCHF) among medical and pharmacy students of Pakistan. BMC Public Health. (2018) 18:1333. 10.1186/s12889-018-6248-1 30509226 PMC6276267

[21] Ahmed A Saqlain M Tanveer M. Knowledge, attitude and perceptions about crimean congo haemorrhagic fever (CCHF) among occupationally high-risk healthcare professionals of Pakistan. BMC Inf Dis. (2021) 21:1–9. 10.1186/s12879-020-05714-z PMC7792042 33413164

[22] Abdelhafiz AS Mohammed Z Ibrahim ME Ziady HH Alorabi M Ayyad M. Knowledge, perceptions, and attitude of egyptians towards the novel coronavirus disease (COVID-19). J Comm Health. (2020) 45:881–90. 10.1007/s10900-020-00827-7 32318986 PMC7173684

[23] Zhong B-L Luo W Li H-M Zhang QQ Liu XG Li WT. Knowledge, attitudes, and practices towards COVID-19 among Chinese residents during the rapid rise period of the COVID-19 outbreak: a quick online cross-sectional survey. Int J Biol Sci. (2020) 16:1745. 10.7150/ijbs.45221 32226294 PMC7098034

[24] Wolf MS Serper M Opsasnick L O'Conor RM Curtis L Benavente JY. Awareness, attitudes, and actions related to COVID-19 among adults with chronic conditions at the onset of the US outbreak: a cross-sectional survey. Ann Int Med. (2020) 173:100–9. 10.7326/M20-1239 32271861 PMC7151355

[25] Sohrabi C Alsafi Z O'Neill N Khan M Kerwan A Al-Jabir A. World Health Organization declares global emergency: a review of the 2019 novel coronavirus (COVID-19). Int J Surgery. (2020) 76:71–6. 10.1016/j.ijsu.2020.02.034 PMC7105032 32112977

[26] Uphoff EP Newbould L Walker I Ashraf N Chaturvedi S Kandasamy A. A systematic review and meta-analysis of the prevalence of common mental disorders in people with non-communicable diseases in Bangladesh, India, and Pakistan. J Glob Health. (2019) 9:020417. 10.7189/jogh.09.020417 31893031 PMC6925965

[27] Nawaz A Su X Barkat MQ Asghar S Asad A Basit F. Epidemic spread and its management through governance and leadership response influencing the arising challenges around COVID-19 in Pakistan—a lesson learnt for low income countries with limited resource. Front Public Health. (2020) 8:573431. 10.3389/fpubh.2020.573431 33363079 PMC7758222

[28] Jahangir R. Internet Use Sees Sharp Spike. (2020). Available online at: https://www.dawn.com/news/154407931 (accessed May 31, 2020).

[29] Wåhlberg AA Sjöberg L. Risk perception and the media. J Risk Res. (2000) 3:31–50. 10.1080/136698700376699

[30] Hayat K Rosenthal M Xu S Arshed M Li P Zhai P. View of Pakistani residents toward coronavirus disease (COVID-19) during a rapid outbreak: a rapid online survey. Int J Environ Res Public Health. (2020) 17:3347. 10.3390/ijerph17103347 32408528 PMC7277197

[31] Azlan AA Hamzah MR Sern TJ Ayub SH Mohamad E. Public knowledge, attitudes and practices towards COVID-19: A cross-sectional study in Malaysia. PLoS One. (2020) 15:e0233668. 10.1371/journal.pone.0233668 32437434 PMC7241824

[32] Ullah I Khan KS Tahir MJ Ahmed A Harapan H. Myths and conspiracy theories on vaccines and COVID-19: potential effect on global vaccine refusals. Vacunas. (2021). 10.1016/j.vacun.2021.01.001. [Epub ahead of print]. PMC7951562 33727904

[33] Khan S Gilani US Raza SMM Hussain T. Evaluation of general awareness among professionals regarding COVID-19: a survey based study from Pakistan. Res Square. (2020) (Under Review).

[34] Fegert JM Vitiello B Plener PL Clemens V. Challenges and burden of the Coronavirus 2019 (COVID-19) pandemic for child and adolescent mental health: a narrative review to highlight clinical and research needs in the acute phase and the long return to normality. Child Adol Psychiatry Mental Health. (2020) 14:1–11. 10.1186/s13034-020-00329-3 32419840 PMC7216870

[35] Alahdal H Basingab F Alotaibi R. An analytical study on the awareness, attitude and practice during the COVID-19 pandemic in Riyadh, Saudi Arabia. J Inf Public Health. (2020) 13:1446–52. 10.1016/j.jiph.2020.06.015 32563674 PMC7832465

